# Data, and sample sources thereof, on water quality life cycle impact assessments pertaining to catchment scale acidification and eutrophication potentials and the benefits of on-farm mitigation strategies

**DOI:** 10.1016/j.dib.2022.108505

**Published:** 2022-08-02

**Authors:** Graham A. McAuliffe, Yusheng Zhang, Adrian L. Collins

**Affiliations:** Net Zero and Resilient Farming, Rothamsted Research, North Wyke, Okehampton, Devon EX20 2SB, UK

**Keywords:** Agriculture, system analysis, Geographical information systems, Environment, Big data

## Abstract

Based on recent spatially aggregated June Agriculture Survey data and site-specific environmental data, information from common farm types in the East of England was sourced and collated. These data were subsequently used as key inputs to a mechanistic environmental modelling tool, the Catchment Systems Model, which predicts environmental damage arising from various farm types and their management strategies. The Catchment Systems Model, which utilises real-world agricultural productivity data (samples and appropriate consent provided within the Mendeley Data repository) is designed to assess not only losses to nature such as nitrate, phosphate, sediment and ammonia, but also to predict how on-farm intervention strategies may affect environmental performance. The data reported within this article provides readers with a detailed inventory of inputs such as fertiliser, outputs including nutrient losses, and impacts to nature for 1782 different scenarios which cover both arable and livestock farming systems. These 1782 scenarios include baseline (i.e., no interventions), business-as-usual (i.e., interventions already implemented in the study area) and optimised (i.e., best-case scenarios) data. Further, using the life cycle assessment (LCA) methodology, the dataset reports acidification and eutrophication potentials for each scenario under two (eutrophication) and three (acidification) impact assessments to offer an insight into the importance of impact assessment choice. Finally, the dataset also provides its readers with percentage changes from baseline to best-case scenario for each farm type.

## Specifications Table

**Table:** 

Subject	Hydrology and Water Quality
Specific subject area	Spatially-explicit life cycle assessment in the context of agricultural water pollution
Type of data	Tabular dataset
How the data were acquired	The data used for the Catchment Systems Model (CSM) [1] and subsequent LCA modelling were adopted from several sources. For farm structures including cropping areas, livestock populations, etc., data were based on individual farm surveys undertaken by Defra (the UK Department for Environment, Food and Rural Affairs [2]. These strategic surveys were supplemented by additional data collection using a simple pro forma (five samples, of which are provided herein) to illustrate the type of data that underlies the CSM model. Following data collection and model development, information was aggregated to Water Framework Directive (WFD) waterbodies and finally disaggregated by Robust Farm Types (RFTs) for the present study. Farm type specific fertiliser applications for different crops were based on the British Survey of Fertiliser Practice (BSFP, https://www.gov.uk/government/collections/fertiliser-usage). Long term average (1981–2010) annual rainfall was estimated based on the HadUK-Grid data which provides a collection of gridded climate variables derived from the network of UK land surface observations at 1 km x 1 km resolution [3]. The soil drainage status was inferred from NatMap 1000 (http://www.landis.org.uk/data/nm1000.cfm) which lists relevant soil series for each 1 km x 1 km cell. Previously developed pedo-transfer functions [1] were used to determine soil drainage status for the relevant soil series.
Data format	Raw Analyzed
Description of data collection	Data were collected and subsequently anonymised to protect farmers’ identities through a national survey and a small supportive survey (five samples are provided in the data repository to give future potential users an idea of what information was collected). Data for major RFTs were combined to broad farm types; namely, arable farms and livestock farms. The former includes cereals, general cropping, horticulture and the latter includes lowland grazing and dairy. These farm types were then combined with the spatial patterns of annual rainfall and soil drainage status to create pseudo farms which represented arable and livestock farm types covering each water catchment within the study area.
Data source location	• Institution: Rothamsted Research • City/Town/Region: Hertfordshire • Country: England • Latitude and longitude (and GPS coordinates, if possible) for collected samples/data: N/A due to anonymised nature of the data • Impact assessments: ReCiPe [4]; Centre for Environmental Studies (CML) [5]; Environmental Product Declarations (EPD) [6].
Data accessibility	Repository name: Mendeley Data Data identification number: DOI: 10.17632/wry3659sjw.4 Direct URL to data: https://data.mendeley.com/datasets/wry3659sjw/draft?a=49462c9f-e549-46f0-9788-a74d813ec1a8[7].
Related research article	G.A. McAuliffe, Y. Zhang, and A.L. Collins. Assessing catchment scale water quality of agri-food systems and the scope for reducing unintended consequences using spatial life cycle assessment (LCA). Journal of Environmental Management*.* 318 (2022) 115563. https://doi.org/10.1016/j.jenvman.2022.115563[8].

## Value of the Data

• Life cycle assessment is typically carried out without spatial visualisation of impact assessments· This dataset provides water quality impacts of over 1700 farms (both arable and livestock) which vary from no mitigation strategies to multiple mitigation strategies and demonstrates the benefits of on-farm interventions. The dataset, whilst demonstrating a novel method rather than being a full case-study, could be used by farmers and policymakers to identify where various farming interventions could be deployed geographically to maximise the best management of farming in terms of damage to nature · These data provide a first step to build upon through out-scaling of spatial life cycle assessment to cover water catchments and their potential for water quality improvements, thus enabling stakeholders to target areas where improvements in water quality, or indeed reductions in other environmental impacts, should be prioritised.

## 1 Data Description

The dataset comprises 1782 unique farms differentiated by location, intervention (or lack thereof), losses to nature associated with said interventions, and finally their impact assessments. Whilst not all data are used in the main paper, they are reported herein for transparency. For example, soil types 0, 1, and 2 refer to free draining, drained for arable and drained for arable and grass, respectively. Numbers 2 and 3 under rainfall, on the other hand, refer to 600–700 mm and 700–900 mm of rainfall per annum, respectively. Each farm ID refers to (a) its location (geographical identification anonymised for farmer protection) and (b) whether it is a predominately arable (1–24) or predominately livestock (25–39) farm. Despite this anonymisation, we have provided end-users with five completed sample surveys for additional transparency, in addition to a consent form which we received from the five participating farmers. Please note that these survey samples are not taken directly from the national June Agriculture Survey (JAS) [3] and are, instead, in-house designed templates to sense-check the validity of licensed (i.e., we do not have permission to share) government survey data. The area for each farm system type calculated under the CSM framework is reported in Column E of the LCI/LCIA dataset, which acts as the functional unit for scaling LCA impact assessments (i.e., acidification potential and eutrophication potentials reported under multiple impact methods). Columns F-K predict the amount of losses to the environment for a range of pollutants for each farm type, whilst Column L reports the carbon stock as tonnes/ha. Energy use (as diesel) is reported in Column M, whilst columns N-P provide the amount of fertiliser used on each farm. Columns Q-Z present the impact assessment results for each farm with Row 1 providing detailed information on the methods used to derive said impacts. Each survey file (i.e., ‘Farm 1–5’) provides an insight, although incomplete due to licensing reasons outlined above, to demonstrate how data were cross-checked prior to modelling integration within the CSM framework, which ultimately provided the LCI data for the spatial LCIA.

## 2 Experimental Design, Materials and Methods

The dataset provided on Mendeley Data was designed using a combination of process-based models (the Catchment Systems Model), deterministic modelling (life cycle assessment) and spatial analysis (Geographical Information Systems). The hypothesis of the study was to test the efficacy of reporting high-resolution (i.e., catchment scale) agri-environmental life cycle assessments in a spatially relevant manner. Data were first collected through a detailed survey of farmers (again, conducted by Hollis et al. [3], with sample cross-checks provided as complementary assistance to understanding current farm management) to quantify their inputs and outputs and then, subsequently, thousands of scenarios were calculated using the Catchment Systems Model to calculate losses of ammonia, nitrate, nitrous oxide and phosphorus [9], [10], [11], [12], [13]. These calculations were subsequently used as a life cycle inventory to calculate three different impact assessments (ReCiPe, Centre for Environmental Management, known as CML, and Environmental Product declarations, known as EPD). Vertically (i.e., by row), the dataset compares various on-farm interventions versus no interventions (baseline) and business-as-usual (BAU; how farmers in the area currently carry out their activities in the real world). Horizontally (i.e., the right-most columns), the dataset compares how choice of life cycle impact assessment affects the results of the different scenarios.

## Ethics Statement

Hereby, we (Graham A. McAuliffe, Yusheng Zhang, and Adrian L. Collins) have declared that no human subjects and/or animals were used for the reported research. All authors have no known competing financial interests or personal relationships that could have influenced the work reported in this paper. For the limited bespoke farm surveys, consents were obtained from individual respondents for the use of their returns. All returned farm and personal data were held in strict compliance with UK GDPR regulations and care was taken to ensure that individual farm confidentiality and privacy were maintained throughout data acquisition, storage and use.

## CRediT authorship contribution statement

**Graham A. McAuliffe:** Conceptualization, Formal analysis, Investigation, Methodology, Software, Validation, Writing – original draft, Writing – review & editing. **Yusheng Zhang:** Formal analysis, Data curation, Validation, Software, Visualization, Writing – review & editing. **Adrian L. Collins:** Funding acquisition, Project administration, Resources, Supervision, Writing – review & editing.

## Declaration of Competing Interest

The authors declare that they have no known competing financial interests or personal relationships that could have appeared to influence the work reported in this paper.

## Data Availability

Data on water quality life cycle impact assessments pertaining to catchment scale acidification and eutrophication potentials and the benefits of on-farm mitigation strategies (Original data) (Mendeley Data).

## References

[1] Zhang Y., Granger S.J., Semenov M.A., Upadhayay H.R., Collins A.L. (2022). Diffuse water pollution during recent extreme wet-weather in the UK: environmental damage costs and insight into the future?. J. Clean. Prod..

[2] Defra (2021). https://www.gov.uk/government/collections/farm-practices-survey.

[3] Hollis D., McCarthy M., Kendon M., Legg T., Simpson I., Met Office (2018). http://catalogue.ceda.ac.uk/uuid/4dc8450d889a491ebb20e724debe2dfb.

[4] Huijbregts M.A.J., Steinmann Z.J.N., Elshout P.M.F., Stam G., Verones F., Vieira M., Zijp M., Hollander A., van Zelm R. (2017). ReCiPe2016: a harmonised life cycle impact assessment method at midpoint and endpoint level. Int. J. Life Cycle Assess..

[5] CML, CML (2016). https://www.universiteitleiden.nl/en/research/research-output/science/cml-ia-characterisation-factors.

[6] EPD, 2013. Environmental product declaration impact assessment (SimaPro). Swedish Environmental Management Council, Stockholm. https://environdec.com/about-us/global-house-of-epd. Accessed July 16, 2021.

[7] McAuliffe G.A., Zhang Y., A. L (2022). Collins spatial life cycle impact assessment data for catchment scale acidification and eutrophication potentials. Mendeley Data.

[8] McAuliffe G.A., Zhang Y., Collins A.L. (2022). Assessing catchment scale water quality of agri-food systems and the scope for reducing unintended consequences using spatial life cycle assessment (LCA). J. Environ. Manag..

[9] Webb J., Misselbrook T.H. (2004). A mass-flow model of ammonia emissions from livestock production. Atmos. Environ..

[10] Lord E.I. (1992). Modeling of nitrate leaching: nitrate sensitive areas. Asp. Appl. Biol..

[11] Lord E., Anthony S. (2000). MAGPIE: a modelling framework for evaluating nitrate losses at national and catchment scales. Soil Use Manag..

[12] Stromqvist J., Collins A.L., Davison P.S., Lord E.I. (2008). PSYCHIC – a process-based model of phosphorus and sediment transfers within agricultural catchments. Part 2. A preliminary evaluation. J. Hydrol..

[13] Collins A.L., Stromqvist J., Davison P.S., Lord E.I. (2007). Appraisal of phosphorus and sediment transfer in three pilot areas identified for the catchment sensitive farming initiative in England: application of the prototype PSYCHIC model. Soil Use Manag..

